# Analysis of quality of life and periodontal health with an eight-unit maxillary fixed retainer through a prospective clinical trial

**DOI:** 10.1038/s41598-025-88309-5

**Published:** 2025-02-05

**Authors:** Mohamed H. Abbas, Essam M. Abdalla, Nadia M. El Harouni, Eiman S. Marzouk

**Affiliations:** Department of Orthodontics, Faculty of Dentistry, Alexandria University, Champollion St, Azarita, P. O. Box: 21521, Alexandria, Egypt

**Keywords:** Extended fixed retainers, PDL, Quality of life, Health care, Medical research

## Abstract

Retention is crucial in orthodontics, aiming to preserve treatment outcomes and enhance patient satisfaction with permanent fixed retainers. However, a removable retainer may be needed with fixed retainers to prevent unwanted changes. An eight-unit extended fixed retainer is proposed to eliminate the need for a removable retainer, addressing the undesired changes associated with six-unit fixed retainers. The impact of extended retainers on periodontium and quality of life remains unexplored. This study evaluates the periodontal response and patient-reported quality of life with an eight-unit maxillary fixed retainer. A single-arm prospective clinical trial with only twenty-eight test group patients (6 males, 22 females) who finished active orthodontic treatment were recruited. The mean age was (19.8 ± 4.5 years). This research was approved by the institutional review board of the Faculty of Dentistry, Alexandria University (IORG:0008839, No-0479-8/2022). The registration date of this study was (5/06/2023). An eight-unit maxillary fixed retainer was bonded to the palatal surface of the maxillary incisors, canines, and first or second premolars directly after debonding the brackets. The periodontal assessment and quality of life were carried out through clinical examination and valid questionnaires and the patients were followed up for 12 months. The periodontal response improved significantly at 1-year follow-up. The Probing depth, Gingival index, Plaque index, Bleeding index, and mobility index were significantly lower in these patients. Quality of life was assessed by the OHIP-14. The mean score decreased by -1.86 (SD = 4.19), and the acceptance of the orthodontic appliance scale score increased by 0.68 (SD = 0.86), which was significant with a p-value < 0.001. For 12 months of follow-up, an eight-unit maxillary retainer did not adversely affect the periodontal ligaments. meets patients’ expectations and maintains a high quality of life.

## Introduction

Orthodontic treatment involves the correction of malocclusion. Once the treatment ends, a retention period starts to maintain the corrected results^1,2^ Without retention, teeth tend to shift back to their original position which can be termed (relapse)^3,4^. Commonly used means of retention in orthodontics include removable retainers and fixed retainers (FRs)^4^. Many removable retainers have been used. They were shown to be effective at retaining teeth to their final position, yet they are compliance-demanding appliances^1^.

Fixed retainers are commonly chosen by orthodontists due to their aesthetic appeal and ability to ensure retention without relying on patient compliance, while 11% favor them in the maxillary arch^5^.

Several studies^4,6–12^ have shown that unwanted changes in tooth position are not related to the original malocclusion, and these changes are associated with canine-to-canine FR alone (6-unit).

These changes cannot be termed relapse, but rather unwanted changes. The exact reason for these unwanted changes remains unclear^13^. These movements, even if FR is in place, range from minor rotations for individual teeth to rotations of the whole segment connected with the FR, with a fulcrum in the lower incisors^13–15^.

A trend toward dual retention instead of solitary removable or fixed retention is more frequently used to avoid the side effects of canine-to-canine fixed retainers alone. however, this trend is still dependent on the patient’s compliance^16–18^. An extended fixed retainer would be a simpler alternative to dual retention protocols for overcoming the drawbacks of canine-to-canine retainers. The extended fixed retainer was tested only in the mandibular arch in extraction cases^19^. The extended retainer was effective at preserving extraction spaces and maintaining results during retention with no unwanted changes in tooth position connected by FR^19^.

Studies on extended maxillary fixed retainers are scarce in the literature, especially regarding the extent of extension of the FR, the extent of changes in tooth position associated with the FR, the impact of the FR on periodontal tissues especially in the maxillary arch, and patient response along with quality of life.

The effects of FR on periodontal health were previously investigated^20–25^. several studies^26,29,31^ reported that FR did not have any significant negative effects on periodontal health. Other studies have shown potential harm to the periodontal ligament (PDL). However, most of these studies indicate that the negative effects are related primarily to soft tissues rather than hard tissues^26,27^.

The effects of orthodontic retainers on speech, self-esteem, and quality of life were investigated. It was reported that temporary speech problems were commonly reported after patients received retainers, these problems lasted from a few days to a few weeks up to 3 months^28–30^.

This adjustment period can potentially impact patient self-esteem and quality of life, leading to noncompliance with removable retainers and potential relapse. Krämer^28^, reported that patients wearing FRs had lower levels of pain, discomfort, soreness, and tension than those wearing Essix retainers. Patients with FR also found it easier to adjust to the retainer. Similarly, Al-Moghrabi^32^ concluded that FRs cause less discomfort and speech difficulties and require less patient compliance. A systematic review^30^ concluded that Essix retainers should be avoided if patient compliance is desired, recommending FRs as an alternative. Thus, FRs are recommended for better speech function, aesthetics, stability, and overall quality of life.

An extended fixed retainer was hypothesized to be a simpler alternative to dual retention protocols while attempting to overcome canine-to-canine FR drawbacks since the number of units to be bonded is increased^19^. The extended fixed retainer was tested only in the mandibular arch in extraction cases, although the unwanted movement was found to be more strongly associated with maxillary fixed retainers^19,31^. There is a lack of studies on extended maxillary fixed retainers in the literature, regarding the extension of the FR, the nature of the associated changes in tooth position, and effects on periodontium and quality of life.

Therefore, in this study, aiming to study the possibility of eliminating the need for additional removable retainers (dual retention), an eight-unit extended maxillary fixed retainer was bonded directly after finishing the active orthodontic phase to assess PDL response and patients’ quality of life.

## Methods

This study was conducted to assess the periodontal response to an eight-unit extended maxillary fixed retainer without removable retainers, along with quality of life, and patient satisfaction assessment.

This research was approved by the institutional review board of the Faculty of Dentistry, at Alexandria University. all methods were carried out in accordance with ethical approval to conduct research on human subjects follows the Declaration of Helsinki(IORG:0008839, No-0479-8/2022).

All experimental protocols were approved by institutional review board of the Faculty of Dentistry, at Alexandria University and ethics committee .

The entire study was conducted at the Orthodontic Department at Alexandria University. The first trial registration date of this study was (5/06/2023) following all ethical considerations of Clinical trials.

An informed consents to all patients who had been selected to that research and/or their legal guardian(s).

This research was a single-center prospective interventional open-label single-arm clinical trial, and the study was registered at Clinicaltrials.gov (NCT05889884).

### Inclusion criteria

Patients who had just finished the orthodontic fixed appliance phase (at least one year of treatment) with extraction or non-extraction treatment and scheduled to start retention.

### Exclusion criteria

Patients with active periodontal disease^32^, systemic disease, bone disease, craniofacial syndromes, cleft, active transverse palatal expansion, malformation, abnormal surface or morphological tooth structure, or restorations were excluded from the study.

### Sample size calculation

The sample size was estimated assuming a 5% alpha error and 80% study power. The sample size was adjusted to a 95% confidence interval (95% CI) to detect changes in probing depth after fixed retainer use. Salvesen et al.^33^ reported a mean (SD) probing depth of 2.9 (0.7) mm with a calculated 95% confidence interval = 2.41, 2.99. The required sample size was calculated to be 25 patients, which was increased to 28 to compensate for patients lost to follow-up. The sample size was calculated using MedCalc Statistical Software version 19.0.5 (MedCalc Software bvba, Ostend, Belgium; https://www.medcalc.org; 2019).

### Patient preparation

First, the study procedures were thoroughly explained to both the participants and their parents and, informed consent was subsequently obtained from each enrolled subject.

At the T0 baseline, patients had phase one nonsurgical periodontal therapy (full mouth supragingival and subgingival scaling, root planing and polishing with eugenol-free paste followed by proper oral hygiene instructions (using a toothbrush, dental floss, and interdental brush) before bonding the FR.

### Intervention

At T0 (after bracket debonding, and just before bonding the FR) a full PDL assessment was carried out for the maxillary dentition including (the probing depth, bleeding index, gingival index, mobility index, and plaque index)^35^ An impression was made at T0 to fabricate a removable retainer in the case when significant changes occurred during follow-up, which might necessitate immediate study termination, (Futility point) and the use of this retainer is to restore the T0 state.

### Bonding steps of the extended FR

Several measures have been taken to ensure high bond strength while overcoming the high rate of failure of maxillary FR.

First, pumice polishing was done for all surfaces to be bonded^34^, followed by a sodium hypochlorite swab for 1 min^35^ (Sodium Hypochlorite 5% mint flavor, JK dental vision, Egypt) then acid etching by phosphoric acid 36% for 15 s, along with rinsing etchant surface same amount of time and gentle drying^36^.

**Fig. 1:**
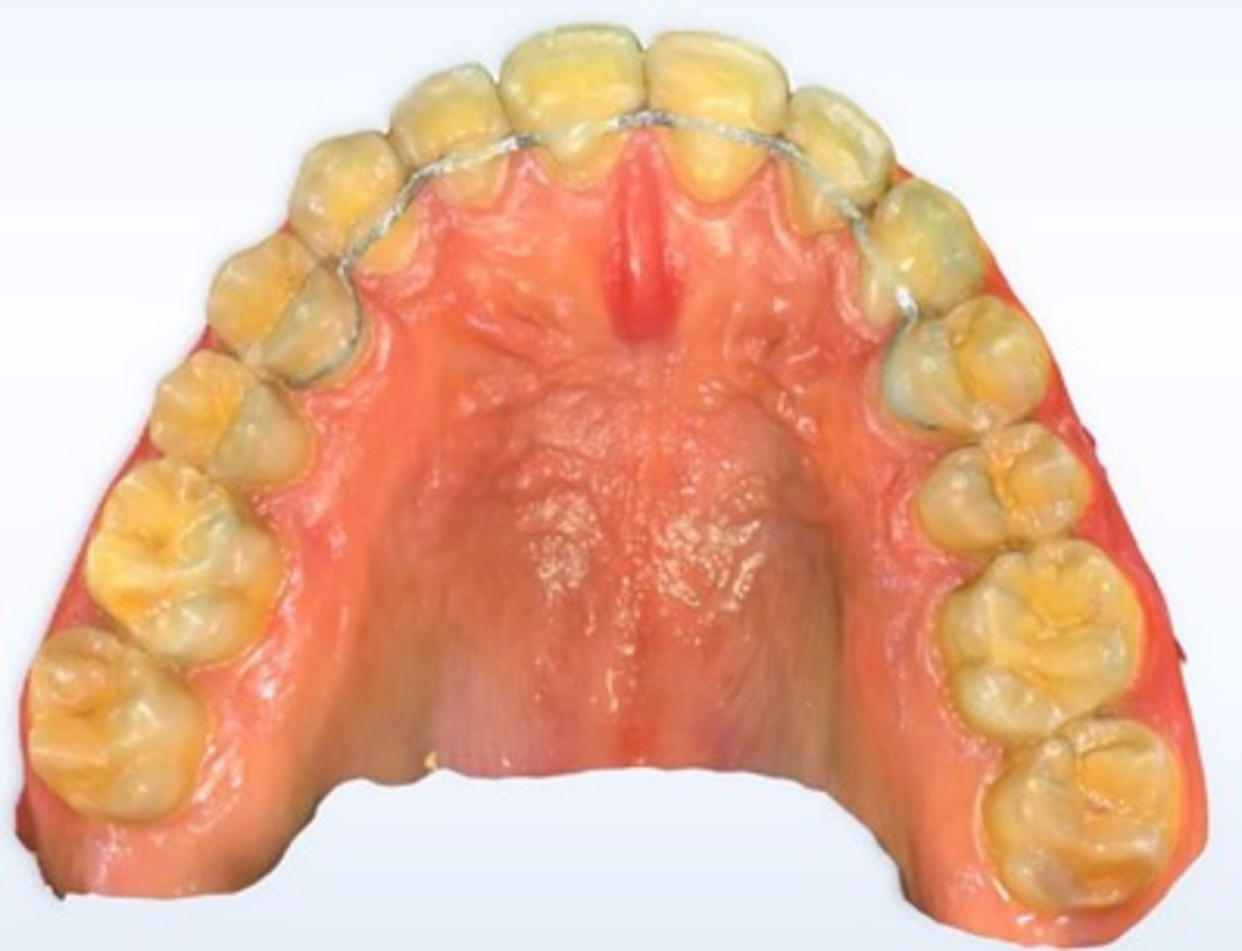
Cropped digital image from an intraoral scan of an eight-unit extended maxillary fixed retainer.

Pre-hydrolyzed no-mix silane primer and Silane coupling agents (BISCO PORCELAIN PRIMER, BISCO, USA) were added to all surfaces to be bonded^37^. The next step was, Bonding agent application (ASSURE® PLUS, Reliance Orthodontic Products, USA). Holding of the FR was done with the help of dental floss then direct adaptation and festooning of Dead soft wire, 0.027 × 0.011-inch ribbon arch-wire, 8-strand braided wire (FR) (Bond-A-Braid® Lingual Retainer Wire, Reliance Orthodontic Products, USA) from the palatal surface of right premolar to left premolar including the palatal surface of all maxillary anterior teeth in passive state away from the line of occlusion. (Fig. 1) The flowable light-curing composite was applied (Polofil® NHT flowable composite light-curing, voco, Germany). Curing for 3 s using high-intensity LED was carried on.( light intensity 2300 mW/cm²)10 W^38^.

After finishing the whole curing for all units, selective grinding of excess composite or any interference between FR and lower teeth was done using articulating paper followed by polishing all composite surfaces eliminating any rough area.

Details of oral hygiene instructions were provided, including a thorough explanation of the flossing technique to be used with the retainer in place, in addition to guidance on utilizing interdental brushes and water flossers.

Patients were given the questionnaires and they filled them out directly after bonding with the FR to assess their quality of life and experience with the extended FR.

Patients were followed up regularly each month and were asked to urgently to schedule an appointment if they felt any detachment in the FR.

After 12 months of follow-up(6) (T1), all the previous records were repeated with periodontal assessment. Patients were given the quality-of-life questionnaires again to fill them.

### Statistical analysis

Normality was checked for all variables using descriptive statistics (mean, median, and standard deviation), plots (Q-Q plots and histogram), and normality tests. Means and standard deviations were calculated for quantitative variables, while frequencies and percentages were calculated for qualitative variables. Comparisons of quantitative variables at T0 and T1 were performed using paired t-test for normally distributed variables and Wilcoxon signed rank test for non-normally distributed variables. The mean difference and 95% confidence intervals (CIs) were calculated. Comparisons of qualitative variables at T0 and T1 were performed using the McNemar test. A p-value < 0.05 indicated statistical significance. The data were analyzed using IBM SPSS for Windows (version 26.0).

### Outcome assessment

#### Periodontal indices

The probing depth was defined as the distance from the gingival margin to the base of the sulcus or periodontal pocket within a normal range of (1–3 mm).

The bleeding Index^38^ was used to evaluate bleeding as follows:

1. Only one bleeding point appears.
2. Several isolated bleeding points or a small blood area appear.
3. Interdental triangle filled with blood soon after probing.
4. Profuse bleeding when probing, blood spreads toward the marginal gingiva.

The mobility index was used to assess mobility by scores as follows^39^:

1. Tooth mobility is perceptible but less than 1 mm buccolingually.
2. Mobility between 1 and 2 mm
3. Mobility exceeds 2 mm buccolingually or vertically

The plaque index was used to assess the amount of plaque on teeth with the aid of plaque-disclosing tablets^40^. (Biofilm Disclosing tablets (EMS) – Guided Biofilm Therapy, Biofilm Disclosure).

0. No plaque in the gingival area.
1. Separate flecks of plaque at the cervical margin of the tooth.
2. A thin continuous band of plaque (up to 1 mm) at the cervical margin.
3. Abundance of soft matter within the gingival pocket and/or on the tooth and gingival margin.

*The gingival index* was used to assess the condition of the gingiva according to the following score^41^:

0 Normal gingiva with slight color change, and slight edema. There was no change in probing.

1. Mild inflammation
2. Moderate inflammation redness, edema, and glazing. Bleeding on probing.
3. Severe inflammation marked redness, edema, and ulceration. Tendency to spontaneous bleeding.

### Quality of life assessment

**Fig. 2:**
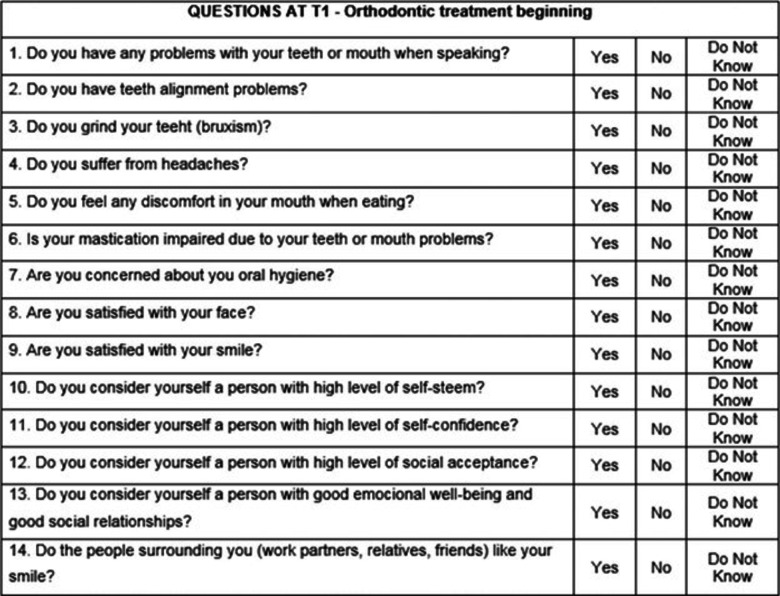
Cropped image for the orthodontic treatment questionnaire.

The orthodontic treatment questionnaire is composed of 14 questions that evaluate the patient’s response (Yes/No/not know) to the method of orthodontic treatment which was the orthodontic retention phase in this research (Fig. 2)

**Fig. 3:**
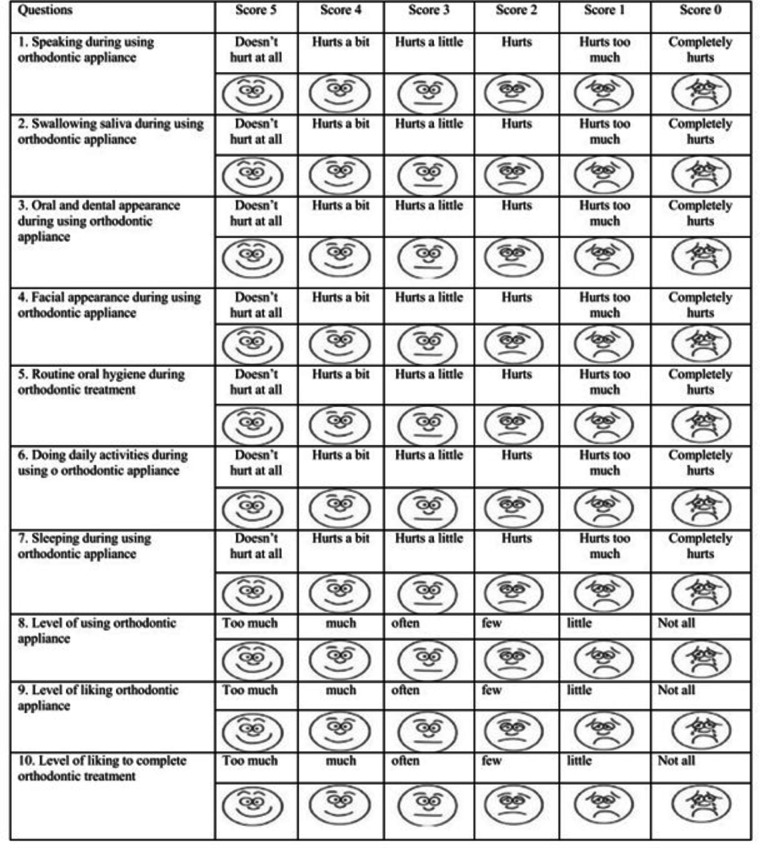
Cropped image for the acceptance of orthodontic appliance questionnaire.

Acceptance of orthodontic appliance scale^42^: This scale consists of 10 incomplete statements, that need to be completed based on the patient’s choice. The available answer choices were scored using a 6-point Likert scale. To help patients understand the answer items, each answer was accompanied by a matching facial expression. Scores of 5 to 0 were allocated to the answer choices from left to right. Higher scores indicate greater acceptance and satisfaction with the respective item. The total score of this questionnaire ranged from 0 to 55. A higher total score indicated that the problems associated with using the removable orthodontic appliance were better accepted by the patient and reflected privileged motivation to continue the treatment. (Fig. 3)

**Fig. 4:**
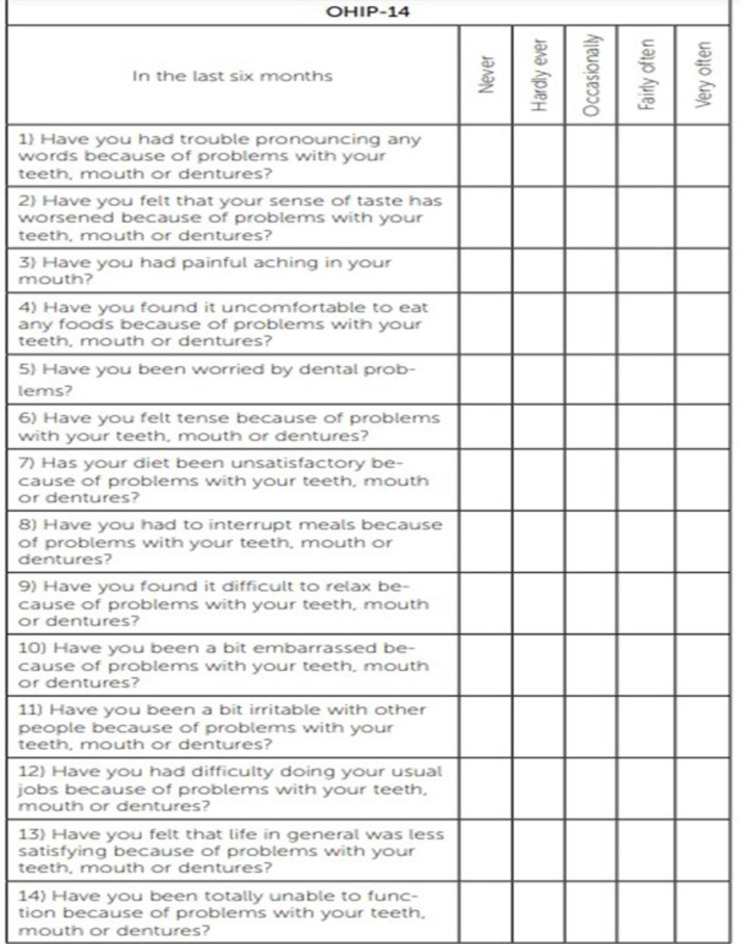
Cropped image for the oral health impact profile-14 questionnaire.

The Oral Health Impact Profile (OHIP-14)^43^ is a valid and reliable instrument for assessing oral health-related quality of life among the adult population. The responses are rated on a 5-point Likert scale: 0 = never; 1 = hardly ever; 2 = occasionally; 3 = fairly often; 4 = very often/every day. The OHIP-14 scores can range from 0 to 56 and are calculated by summing the ordinal values for the 14 items. (Fig. 4)

## Results

The demographic data for all patients (*n* = 28) are shown in Table 1. All PDL indices were measured by two different examiners. Interexaminer reliability was calculated and the intraclass correlation coefficient (ICC) ranged from 0.940 to 0.999, indicating good to excellent agreement between the examiners. ^44^

**Table 1:** Sample description and distribution (*n* = 28).

Age	Mean (SD)	19.82 (3.33)
Gender: n (%)	Male	6 (21.4%)
	Female	22 (78.6%)
Treatment technique: n (%)	Extraction	11 (39.3%)
	Non-extraction	17 (60.7%)

*SD* standard deviation, n: frequency.

All the periodontal indices shown in Table 2, for the 8 maxillary teeth bonded to the FR significantly improved after 12 months of follow-up.

**Table 2:** Periodontal response of maxillary teeth bonded to the extended maxillary FR (8 units).

		Pre retention (T0)	Post retention (T1)	Difference (T1-T0)	95% CI	P value
		Mean (SD)				
Probing depth^a^ (mm)	Buccal	2.95 (0.56)	2.14 (0.25)	− 0.81 (0.47)	− 0.99, − 0.63	< 0.001*
	Palatal	3.04 (0.44)	2.14 (0.25)	− 0.89 (0.36)	− 1.03 − 0.75	< 0.001*
	Average	**2.99** (0.49)	**2.14** (0.25)	− 0.85 (0.40)	− 1.01, − 0.70	< 0.001*
Gingival index ^b^	Buccal	1.25(0.54)	0.45 (0.42)	− 0.80 (0.33)	− 0.93, − 0.67	< 0.001*
	Palatal	1.29 (0.56)	0.48 (0.38)	− 0.81 (0.33)	− 0.94, − 0.69	< 0.001*
	Average	**1.27** (0.54)	**0.47** (0.38)	− 0.81 (0.28)	− 0.92, − 0.70	< 0.001*
Plaque index^a^	Buccal	2.00 (0.54)	0.97 (0.39)	− 1.03 (0.47)	− 1.22, − 0.85	< 0.001*
	Palatal	1.98 (0.52)	1.02 (0.44)	− 0.97 (0.49)	− 1.16, − 0.77	< 0.001*
	Average	**1.99** (0.51)	**0.995** (0.37)	− 1.00 (0.46)	− 1.18, − 0.82	< 0.001*
Bleeding index^a^	Buccal	1.59 (0.65)	0.69 (0.50)	− 0.90 (0.36)	− 1.03, − 0.76	< 0.001*
	Palatal	1.58 (0.60)	0.69 (0.49)	− 0.89 (0.41)	− 1.05, − 0.73	< 0.001*
	Average	**1.59** (0.61)	**0.69** (0.45)	− 0.89 (0.32)	− 1.02, − 0.77	< 0.001*
Mobility^b^	Buccal	0.21 (0.24)	0.009 (0.05)	− 0.21 (0.21)	− 0.29, − 0.12	< 0.001*
	Palatal	0.21 (0.24)	0.009 (0.05)	− 0.21 (0.21)	− 0.29, − 0.12	< 0.001*
	Average	**0.21** (0.24)	**0.009** (0.05)	− 0.21 (0.21)	− 0.29, − 0.12	< 0.001*

*SD* standard deviation, *CI* confidence interval. ^a^Paired samples t-test, ^b^Wilcoxon signed ranks test. *Statistically significant at p-value < 0.05. Significant values are in bold.

The index that showed the most significant changes was probing depth. Both the buccal and palatal measurements showed a substantial decrease in probing depth, with mean decreases of -0.81 mm *P* < 0.001, CI(-0.99, -0.63) and − 0.89 mm *p* < 0.001 CI(-1.03, -0.75) respectively. The average probing depth also showed a significant reduction of -0.85 mm *p* < 0.001, CI (-1.01, -0.70). The whole maxillary dentition, also showed significant improvement as shown in Table 3.

**Table 3:** Periodontal response to a fixed retainer (12 units) (maxillary dentition).

		Pre-retention (T0)	Post-retention (T1)	Difference (T1-T0)	95% CI	P value
		Mean (SD)				
Probing depth^a^	Buccal	3.06 (0.55)	2.26 (0.26)	− 0.80 (0.49)	− 1.00, − 0.61	< 0.001*
	Palatal	3.16 (0.45)	2.25 (0.29)	− 0.91 (0.38)	− 1.06, − 0.76	< 0.001*
	Average	3.11 (0.48)	2.25 (0.27)	− 0.86 (0.42)	− 1.02, − 0.69	< 0.001*
Gingival index^b^	Buccal	1.45 (0.52)	0.66 (0.42)	− 0.78 (0.32)	− 0.91, − 0.66	< 0.001*
	Palatal	1.49 (0.53)	0.69 (0.37)	− 0.80 (0.29)	− 0.91, − 0.68	< 0.001*
	Average	1.47 (0.52)	0.68 (0.37)	− 0.79 (0.26)	− 0.89, − 0.69	< 0.001*
Plaque index^a^	Buccal	2.18 (0.46)	1.19 (0.39)	− 0.99 (0.38)	− 1.14, − 0.85	< 0.001*
	Palatal	2.12 (0.49)	1.20 (0.43)	− 0.92 (0.46)	− 1.10, − 0.74	< 0.001*
	Average	2.14 (0.46)	1.19 (0.38)	− 0.95 (0.40)	− 1.11, − 0.80	< 0.001*
Bleeding index^a^	Buccal	1.81 (0.64)	0.89 (0.45)	− 0.92 (0.34)	− 1.05, − 0.79	< 0.001*
	Palatal	1.81 (0.64)	0.89 (0.47)	− 0.92 (0.40)	− 1.07, − 0.76	< 0.001*
	Average	1.81 (0.62)	0.89 (0.43)	− 0.92 (0.32)	− 1.04, − 0.80	< 0.001*
Mobility^b^	Buccal	0.15 (0.17)	0.006 (0.03)	− 0.14 (0.15)	− 0.20, − 0.08	< 0.001*
	Palatal	0.15 (0.17)	0.006 (0.03)	− 0.14 (0.15)	− 0.20, − 0.08	< 0.001*
	Average	0.15 (0.17)	0.006 (0.03)	− 0.14 (0.15)	− 0.20, − 0.08	< 0.001*

*SD* standard deviation, *CI* confidence Interval. ^a^Paired samples t-test, ^b^Wilcoxon signed ranks test. *Statistically significant at p-value < 0.05.

For the quality-of-life assessment, the OHIP-14 total score was used. Table 4 shows that the mean score at T0 was 4.07 ± 4.60 and it decreased to 2.21 ± 2.57 at T1. This difference was statistically significant (*p* = 0.04).

**Table 4:** Quality of life and acceptance of orthodontic treatment scores pre-retention and 12 months in retention.

		Pre retention (T0)	Post retention (T1)	Difference (T1-T0)	*P* value
OHIP-14 total score	Mean (SD)	4.07 (4.60)	2.21 (2.57)	− 1.86 (4.19)	0.04*
	Median (IQR)	2.00 (1.25, 4.00)	2.00 (2.00, 2.00)	0.00 (− 3.00, 0.75)	
Acceptance of orthodontic appliance scale	Mean (SD)	49.25 (0.80)	49.93 (0.26)	0.68 (0.86)	< 0.001*
	Median (IQR)	49.00 (0.80)	50.00 (5.00, 50.00)	0.50 (0.00, 1.00)	

*SD* standard deviation, *IQR* interquartile range. *Statistically significant at p-value < 0.05.

The median score at T0 was 2.00 (IQR = 1.25, 4.00), and it remained the same at T1, with a median of 2.00 (IQR = 2.00, 2.00). The difference in medians was 0.00 (IQR=-3.00,0.75).

For the acceptance of the orthodontic appliance scale, the mean score at T0 was 49.25 (SD = 0.80), and it increased slightly to 49.93 (SD = 0.26) at T1. The difference between T1 and T0 was statistically significant with a p-value < 0.001.

The median score at T0 was 49.00 (IQR = 0.80) and it increased to 50.00 (IQR = 5.00, 50.00) at T1. The difference in medians was 0.50 (IQR = 0.00, 1.00).

Table 5 shows that most of the participants experienced no negative effects on speech, function, or quality of life during the retention period. For example, there were no reported problems with teeth or mouth when speaking post-retention, and all participants reported face and smile satisfaction.

**Table 5:** Orthodontic treatment questionnaire scores pre-retention and 12 months in retention.

	Pre retention			Post retention			*P* value
	Yes	No	Do not know	Yes	No	Do not know	
	*N* %						
Problems with teeth or mouth when speaking	0 (0%)	28 (100%)	0 (0%)	0 (0%)	28 (100%)	0 (0%)	1.00
Teeth alignment problems	0 (0%)	25 (89.3%)	3 (10.7%)	0 (0%)	28 (100%)	0 (0%)	0.25
Bruxism (teeth grinding)	1 (3.6%)	8 (28.6%)	19 (67.9%)	0 (0%)	26 (92.9%)	2 (7.1%)	< 0.001*
Headache	15 (53.6%)	12 (42.9%)	1 (3.6%)	0 (0%)	27 (96.4%)	1 (3.6%)	0.13
Discomfort when eating	4 (14.3%)	24 (85.7%)	0 (0%)	0 (0%)	28 (100%)	0 (0%)	0.50
Impaired mastication	0 (0%)	28 (100%)	0 (0%)	0 (0%)	28 (100%)	0 (0%)	1.00
Oral hygiene concerns	28 (100%)	0 (0%)	0 (0%)	28 (100%)	0 (0%)	0 (0%)	1.00
Satisfaction with face	28 (100%)	0 (0%)	0 (0%)	28 (100%)	0 (0%)	0 (0%)	1.00
Satisfaction with smile	26 (92.9%)	0 (0%)	2 (7.1%)	28 (100%)	0 (0%)	0 (0%)	1.00
High self-esteem	12 (42.9%)	0 (0%)	16 (57.1%)	28 (100%)	0 (0%)	0 (0%)	< 0.001*
High self-confidence	11 (39.3%)	1 (3.6%)	16 (57.1%)	28 (100%)	0 (0%)	0 (0%)	< 0.001*
High social acceptance	12 (42.9%)	1 (3.6%)	15 (53.6%)	28 (100%)	0 (0%)	0 (0%)	< 0.001*
Good emotional well-being and social relationships	9 (32.1%)	1 (3.6%)	18 (64.3%)	28 (100%)	0 (0%)	0 (0%)	< 0.001*
Surrounding people like your smile	8 (28.6%)	0 (0%)	20 (71.4%)	28 (100%)	0 (0%)	0 (0%)	< 0.001*

*Statistically significant at p-value < 0.05.

There were also significant improvements in self-esteem, self-confidence, social acceptance, emotional well-being, and relationships. Before retention, only a few participants reported high self-esteem, self-confidence, and social acceptance. However, after retention, nearly all participants reported high levels of these factors.

Additionally, there were no negative effects on tooth alignment or reported discomfort during eating.

## Discussion

The orthodontic fixed retainer has long been considered an indispensable part of orthodontic treatment, ensuring the stability of dental occlusion and preventing relapse.

Several previous studies^4,46–48^ have shown that FR has some negative effects on PDL due to difficulty in maintaining oral hygiene. However, most of these studies have evaluated the effects on the mandibular arch only^4,46–48^ that is why authors picked the maxillary arch to evaluate the PDL response and speech is more affected by maxillary arch.

There is a substantial variation in the histological structure of both maxillary and mandibular mucosa. The palatal mucosa consists of two constant and homogeneous layers; ortho-keratinized squamous epithelium, and lamina propria composed of dense connective tissue. These layers increase the resistance of the palatal mucosa to periodontal diseases such as inflammation propagation, and recession^49^, unlike nonkeratinized mandibular lingual mucosa which is more prone to inflammation propagation especially when there is a plaque retentive area such as the FR^50,51^.

The authors of this study hypothesized that the addition of an extra two units to 6-unit FR would increase the root surface area of the bonded segment resisting unwanted tooth that can occur with a canine-to-canine fixed retainer, even while the retainer is in place movement with less impact on maxillary palatal periodontium.

Since extended maxillary FR showed good stability with clinical insignificant unwanted tooth movement^45^, authors wanted to check its impact on PDL and quality of life.

According to the results of the present study, the periodontal condition of all the maxillary teeth significantly improved after the 12-month follow-up period. These findings highlight that extended FR use has no harmful effects on the periodontium. The positive results obtained in the current study may be attributed to the removal of orthodontic fixed appliances, which could have acted as a local factor that might have compromised the periodontium. In addition, the debonding of the brackets was followed by full-mouth scaling and polishing along with oral hygiene measures.

These findings are consistent with the results of previous studies^11,12,22,52^, which showed that orthodontic fixed retainers were found to be compatible with periodontal health and did not have severe detrimental effects on the periodontium. A long-term follow-up study^34^, revealed that both fixed and removable retainers were associated with similar levels of gingival inflammation. Optimal oral hygiene before, during, and after orthodontic treatment is essential for preventing increased levels of gingival inflammation. Additionally, Han^46^ tested the effects of fixed retainers on periodontally compromised patients. Despite bonding fixed retainers in periodontitis patients, periodontal health was well maintained when supportive periodontal treatment and oral hygiene education were provided. Thus, it is crucial to emphasize the importance of optimal oral hygiene, supportive periodontal treatment, and patient education in maintaining periodontal health.

Teeth stability and decreased mobility were significantly improved by using an extended fixed retainer which was consistent with the findings of Josef Kučera^53^, who concluded that FR reduces the increase in tooth mobility caused by orthodontic treatment to normal levels. The values of tooth mobility after the placement of retainers were within the range of physiologic tooth mobility, thus they can even be used with patients who have mobile teeth as a means of splinting and retention since they have no significant damaging effects on the PDL.

Speech, as a fundamental mode of communication and expression plays a significant role in daily life. Any alterations to speech patterns can have a profound impact on individuals’ interactions, affecting social relationships, confidence, and self-esteem. In most cases, speech problems are temporary with removable retainers, varying in duration from a few days to a few months^54^. These changes are temporary and take a period of adaptation ranging from 1 week to 3 months^55^, which in turn may affect patient self-esteem and quality of life. This may push the patient to be incompliant with the removable retainer causing relapse, in this study results, there were no reported speech alterations or problems.

Quality of life encompasses a broad range of physical, emotional, and social aspects, making it a key indicator of treatment success. Although numerous studies have investigated the impact of orthodontic treatment on quality of life, few have specifically considered the effects of fixed retainers. Gaining insight into how fixed retainers affect various dimensions of daily life, including eating, oral hygiene maintenance, and overall satisfaction, represents an important step toward a more comprehensive understanding of the orthodontic patient experience.

Patient satisfaction, as an endpoint of successful orthodontic treatment, can determine the ultimate perception of treatment outcomes. Despite their established utility for maintaining dental alignment, fixed retainers have been associated with specific inconveniences, such as difficulty with oral hygiene practices and occasional breakages. Evaluating patient satisfaction with fixed retainers, and identifying factors that contribute to a positive or negative patient experience, can guide both clinicians and patients in decision-making processes and treatment planning.

Overall, the data in Table 5 indicate that FR has no negative impact on patient quality of life or acceptance of orthodontic appliances. The reduction in the OHIP-14 scores signifies a decreased burden of oral health problems, which can lead to improved overall well-being and satisfaction. Additionally, the increase in acceptance scores indicates that patients not only adjust well to wearing fixed retainers but also perceive them as valuable components of their orthodontic treatment.

It is important to note that although statistically significant, the observed differences in both quality of life and acceptance of orthodontic appliance scores might be considered relatively small in clinical terms. However, even small changes can be meaningful to patients, and the statistically significant findings highlight the positive impact of fixed retainers on patients’ experiences. Therefore, the extended maxillary fixed retainer can be used effectively in the maxillary arch without compromising PDL condition, splinting PDL-compromised teeth, eliminating the need for the patient compliance to a removable retainer, enhancing patient quality of life, and eliminating any speech problems.

Although these findings provide valuable insights into the effects of FR on the quality of life, it is crucial to interpret them within the context of this study’s limitations. This research only involved the maxillary arch FR- which is mainly responsible for the deterioration of speech – in an attempt to standardize the sample and confounding factors. the sample size and specific patient characteristics may impact the generalizability of the results. Further research with a larger and more diverse population is warranted to confirm and expand these findings. The addition of an extra two units to a 6-unit FR was challenging since a 6-unit FR is known to have a high failure rate^56^. Several precautions were carried on to overcome this problem. To ensure high bond strength several measures have been taken to provide that. Starting with pumice polishing enhances shear bond strength^34^. Followed by Sodium hypochlorite 5% swap for 1 min to all surfaces to be bonded to enhance bond strength and remove organic pellicle of dental plaque^35^. Pre-hydrolyzed silane primer was added to etched surfaces to enhance the bonding strength^37^. Patients were instructed to regularly follow the integrity of the retainer while brushing, avoid any extra hard food that might break the retainer and if any loss of integrity took place, the patient must immediately ask for an appointment to fix it. Despite using several measures to decrease the failure rate, two cases have experienced breakage of the FR, and the patients presented for repair with the same bonding protocol after removal of the composite attached to the broken parts and tooth without compromising retainer integrity.

Future Longer-term longitudinal follow-up studies are needed to test the long-term effects of the extended FR and the generalizability of the results with larger samples. Further studies are needed to compare the effects of the extended FR against dual retention, 6-unit FR alone, and removable retainers alone since this study is only a single arm study and blinding could not have been possible. Studies regarding the failure rate of extended FR are needed.

## Conclusion

After a 12-month follow-up period, with the limitations of the study, an eight-unit extended maxillary FR may not have adverse effects on maxillary periodontium and may be used as a means of retention without compromising PDL.

An eight-unit maxillary retainer can be used as a splint to decrease tooth mobility associated with orthodontic tooth movement.

Fixed retainers maintain high patient quality of life and acceptance of orthodontic appliances and positively influence patient attitudes toward orthodontic treatment.

## Data Availability

The datasets used during the current study are available from the corresponding author upon reasonable request.
